# Understanding the Journey of Human Hematopoietic Stem Cell Development

**DOI:** 10.1155/2019/2141475

**Published:** 2019-05-06

**Authors:** Akhilesh Kumar, Saritha S. D'Souza, Abir S. Thakur

**Affiliations:** Wisconsin National Primate Research Center, University of Wisconsin, Madison, WI 53715, USA

## Abstract

Hematopoietic stem cells (HSCs) surface during embryogenesis leading to the genesis of the hematopoietic system, which is vital for immune function, homeostasis balance, and inflammatory responses in the human body. Hematopoiesis is the process of blood cell formation, which initiates from hematopoietic stem/progenitor cells (HSPCs) and is responsible for the generation of all adult blood cells. With their self-renewing and pluripotent properties, human pluripotent stem cells (hPSCs) provide an unprecedented opportunity to create *in vitro* models of differentiation that will revolutionize our understanding of human development, especially of the human blood system. The utilization of hPSCs provides newfound approaches for studying the origins of human blood cell diseases and generating progenitor populations for cell-based treatments. Current shortages in our knowledge of adult HSCs and the molecular mechanisms that control hematopoietic development in physiological and pathological conditions can be resolved with better understanding of the regulatory networks involved in hematopoiesis, their impact on gene expression, and further enhance our ability to develop novel strategies of clinical importance. In this review, we delve into the recent advances in the understanding of the various cellular and molecular pathways that lead to blood development from hPSCs and examine the current knowledge of human hematopoietic development. We also review how *in vitro* differentiation of hPSCs can undergo hematopoietic transition and specification, including major subtypes, and consider techniques and protocols that facilitate the generation of hematopoietic stem cells.

## 1. Introduction

Hematopoietic stem cell transplantation (HSCT) therapy has been widely used and is considered as a promising treatment for various blood disorders [1]. HSCs are adult stem cells that can differentiate into specialized blood cells that control immune function, homeostasis balance, and response to microorganisms and inflammation [2]. They were initially discovered when mouse bone marrow cells were transplanted into irradiated mice, resulting in the development of a colony of hematopoietic cells, which were traced to originate from differentiated HSCs [3, 4]. This significant identification by Till and McCulloch further propelled research in investigating the characterization, development, and cultivation of HSCs. HSCs can be harvested from peripheral blood, bone barrow, and umbilical cord blood [5]. HSCs can be used in transplantation techniques and efficient therapies for hematological diseases; however, it is currently not possible to generate therapeutically viable HSCs for human patients [6, 7]. Lack of matched human leukocyte antigen (HLA) donors makes it difficult to take advantage of the clinical benefits of HSCT [8, 9]. Even then, the demand for HSCTs is unlikely to subside as synergetic efforts have been made to replenish other sources of HSCs [10]. Several studies have reported successful expansion of HSC populations while many others are focused on generating HSCs from induced pluripotent stem cells (iPSCs).

The successful derivation of hESC line by Thomson's group in 1998 [11] and hiPSC line by Yamanaka's group in 2007 [12] initiated tremendous interest and effort in utilizing hPSCs as a consistent source in generating unlimited blood cells for therapeutic purposes. With *in vitro* development of HSCs from hPSCs, current shortages of blood donors can be overcome with more cell-based treatments. Significant progress has been attained in the recent years in developing systems for hematopoietic differentiation and producing various lineages of blood cells, including lymphoid and myeloid specification from hPSCs [13–15]. However, generation of HSCs, which has been the desired goal of many current researchers in the field of HSC research, has been limited and unsuccessful. This can mainly be attested to the significant complexity of the embryonic hematopoietic system and the lack in knowledge of specific markers in distinguishing the various stages of embryonic blood cell development. To overcome this limitation, understanding and identifying the sequential progenitors and molecular mechanisms that lead to the formation of specific blood lineages are vital. In this review, we start with describing our current understanding of embryonic hematopoiesis, its structure, and how it is vital in serving as a blueprint for hPSC differentiation studies. We focus on novel progress that had been made in identifying and understanding signaling pathways that scaffold and guide hematopoietic specification from hPSCs and further discuss important approaches in the production of engraftable blood cells. In our concluding section, we discuss the utilization of hPSC differentiation in HSC development and the current limitations that are to be overcome in achieving this goal.

## 2. Development

**Figure 1:**
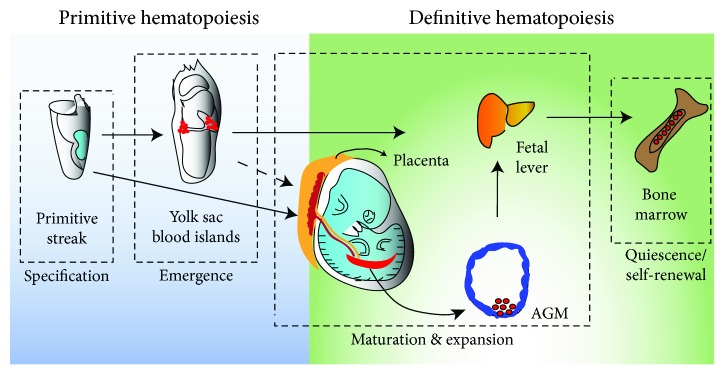
Embryonic hematopoiesis. Establishment of primitive and definitive HSCs during embryonic development.

During development, hematopoiesis occurs in the yolk sac and the embryo proper [16]. However, unlike solid tissues, cells involved in the hematopoietic system are scattered in the organism in different locations [17]. From what is known, HSCs are found in the latter stages of embryogenesis in the major arteries of the embryo, which includes the umbilical and vitelline arteries and the dorsal aorta [18]. Fully developed HSCs can also later be found in the yolk sac and placenta [19]. CD34^+^ cells can be found as early as during week 5 of gestation [20], and most mature HSCs can usually be detected at week 9 of gestation [21, 22]. Once the embryo is fully developed, HSCs then migrate to the fetal liver and expand in the bone marrow for future production and self-renewal during adult life [23]. Since most components of embryonic hematopoiesis have been conserved across multiple species, a general model of the complex development of the hematopoietic system has been established. In the early embryo, many waves of hematopoiesis are initiated and organized in a spatially, temporally, and functionally distinct manner. Initially, the premiere waves of hematopoiesis were described as primitive and definitive hematopoietic waves (Figure 1). The classification of these programs historically was based on the type of erythroblasts that they developed. Erythroblasts that were early emerging, large and nucleated were termed “primitive,” whereas latter erythroblasts in development that are enucleated were termed “definitive.” Recently though, the presence of erythroid, megakaryocyte, and mast cell progenitors, which are known as erythromyeloid progenitors (EMPs), and B-cell and T-cell progenitors, which are known as lymphoid-primed multipotent progenitors (LMPPs), before the emergence of HSCs and the onset of blood circulation has been revealed [24–27]. Despite the presence of EMPs and LMPPs not being reported yet in human hematopoiesis, the detection of EMP and LMPP populations have been observed at E8.25 in the yolk sac of the mouse embryo, following the emergence of primitive hematopoietic progenitors and prior to the detection of HSCs [25]. However though, Keller's group has proposed that these progenitor populations are rather generated by independent programs that are initiated in the yolk sac and are unique from primitive and definitive hematopoiesis [25]. LMPP hematopoiesis includes the lymphoid development of progenitors to B and T cell types that occurs in the yolk sac and also overlaps with EMP hematopoiesis [28, 29]. Through these separate programs, it can be acknowledged that the yolk sac has distinct forms of hematopoiesis during development. An improved understanding of the initiation and regulation of embryonic hematopoiesis will be necessary in identifying lineages that are HSC-dependent and independent.

### 2.1. Primitive Hematopoiesis

Primitive hematopoiesis occurs in the yolk sac and is more restricted, generating cells of only the erythroid, macrophage, and megakaryocytic lineages [30]. Primitive hematopoiesis can also be defined as all blood lineages except HSCs, erythrocytes, and T cells [31]. It is more specified and initiates in blood islands in the mouse embryo (day 7, E7) and human embryo (18-20 days) during the initial gestation period [30] (Figure 1). Erythroblasts derived from the primitive program tend to be larger in size, retain their nuclei, and are surrounded by endothelial cells [30, 32, 33]. Primitive erythroid cells primarily express the embryonic globin genes, which have a higher affinity for oxygen than definitive erythroid cells that are characterized by the exclusive expression of adult forms of *β*-globin [33, 34]. Macrophages and megakaryocytes derived from this stage also exhibit different properties from those derived from the definitive stage. Primitive macrophages have rapid maturation without a monocyte stage during development [35, 36] and megakaryocytes lack an abundance of platelets and have lower ploidy [37, 38]. Further, understanding primitive hematopoiesis unfortunately encounters challenges in identifying *in vitro* differentiation of ESCs and iPSCs with only primitive erythroid precursors available for complete identification [23].

### 2.2. Definitive Hematopoiesis

On the other hand, definitive hematopoiesis occurs after primitive hematopoiesis and has the potential to generate HSCs at different sites involving vasculature. Definitive describes the emergence of hematopoietic progenitors, which produce myeloid, lymphoid, erythroid lineages, and long-term HSCs in the adult organism [39, 40]. This usually occurs in the dorsal aorta in the aorta-gonad-mesonephros (AGM) region of the embryo proper that comprise the aorta, gonads, and mesonephros [40, 41]. The AGM region in the embryo is the main site of definitive hematopoiesis during mid-stage gestation [42–45]. HSCs can also be found in the yolk sac, the placenta, and the head, which has been observed in mouse models [44]. In humans, HSCs can be detected with the expression of vascular and hematopoietic markers like CD34, VE-cadherin, CD117, CD90, CD45, and CD105 [46]. *In vitro*, HSCs that are capable of engraftment can be generated from AGM VE-cadherin^+^ progenitors in a coculture with OP9 stromal cells or endothelial cells [47–50]. Intriguingly, In the AGM region, intra-aortic hematopoietic clusters (IAHCs) can be found on the ventral wall, which signifies the initiation of definitive hematopoiesis in the embryo [51]. These IAHCs cover the endothelial lining of the dorsal aorta and give rise to hematopoietic cells via the transitioning of flat aortic endothelial cells into round hematopoietic cells. It is suggested that specialized hemogenic endothelium in the ventral wall of the dorsal aorta undergo endothelial-to-hematopoietic transition (EHT), giving rise to HSCs. Hence, this suggests that generating hematopoietic cells through endothelial intermediates is a critical step during the development of the hematopoietic system. Additionally, the process of EHT is seen to be conserved across vertebrates, including humans, mice, and zebrafish [39, 52, 53]. Currently, it has been hypothesized that arterial specification is an essential prerequisite for initiating the HSC program and this finding will help in identifying and enhancing lymphomyeloid hematopoietic progenitors and eventually lead to generating engraftable HSCs from hPSC cultures [15, 54]. Earlier, Vo et al. hypothesized that early hematopoietic development during embryogenesis is inhibited by epigenetic silencing [55]. They reported that the Polycomb group protein EZH1 increased the proliferation of lymphoid cells from HSCs and its deficiency in mice results in the early appearance of definitive HSCs in an embryo *in vivo* [55].

## 3. Hemangioblasts

During the late 19^th^ century, embryologists observed a close relationship between endothelial and hematopoietic lineages and later in 1917, Florence Sabin concluded the existence of unique bipotential cells that give rise to blood and endothelial cells based on her experiment on the yolk sac of chicken embryo [56]. The term hemangioblast was coined 15 years later by Murray in reference to a large mass of cells defined as yolk sac mesenchyme from which endothelial and hematopoietic cells develop [57]. Hemangioblasts that develop out of the mesoderm during early embryonic development possess endothelial and hematopoietic properties and are identified as a clonal precursor that can give rise to both blood cells and endothelial cells [58, 59]. Hemangioblasts were subsequently located and observed in the mouse embryo [60], in zebrafish [61], and in *in vitro* differentiating human ESCs [62, 63]. Hemangioblasts are more traced to primitive differentiation predominantly characterized by the coexpression of receptor tyrosine kinase Fl-1/KDR (VEGFR2), the primitive streak transcription factor *Brachyury*, and also by its ability to develop vascular and hematopoietic lineages [60].

## 4. Hemogenic Endothelium (HE)

During hematopoiesis and HSC development, it has been observed that blood cells derive from progenitors that express endothelial properties. These specialized endothelial progenitors known as hemogenic endothelium (HE) are noted to give rise to blood cells through an endothelial-to-hematopoietic transition (EHT) rather than through an asymmetric division [64]. HE is involved in definitive hematopoiesis, and hematopoietic cells are generated newly from this subset of HE [34, 65] which was shown through lineage tracing [66] and time-lapse imaging [39, 62, 64, 67]. HE is more localized and characterized by endothelial-specific markers and morphology and can be found in endothelial layers inside blood vessels. HE expresses endothelial markers VE-cadherin, CD31 [68], c-KIT [69], and transcription factors Runx1 [70] and GATA2 [71].

HE is acknowledged as a significant source of adult-type, mature blood cells that are produced in extraembryonic vasculature that include vitelline, umbilical [72, 73], placental [19], and yolk sac [74–76] vasculature. Though EHT in extraembryonic sites can be observed from HE lining arterial, venous, and capillary vessels [72, 75, 77, 78], HSC potential is only localized in arterial vessels [72]. Most endothelial cells involved in the development of hematopoietic progenitors and HSCs are mostly derived from the aortic endothelial layer and can be traced with KDR (also known as FLK1) expression [51, 79]. While transitioning into HSCs, they begin expressing CD45 in hematopoietic clusters and are highly dependent on Runx1 signaling [80, 81]. These previously mentioned observations provide that blood formation via endothelial intermediates is a critical process in the hematopoietic system and that arterial specification over nonarterial specification of HE can improve and allow for the development of hemogenic and hematopoietic progeny. This observation proved that arterial specification is an essential prerequisite for initiating the definitive hematopoietic program [82].

## 5. Advances in Hematopoietic Differentiation from hPSCs

**Figure 2:**
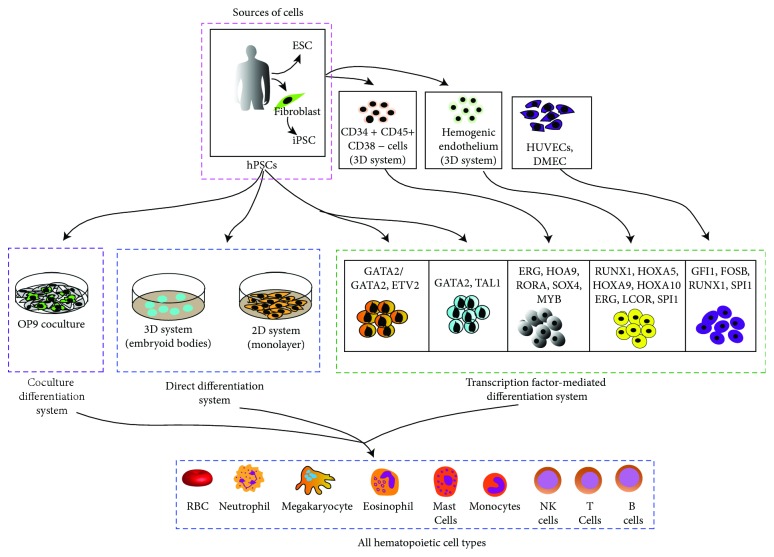
Hematopoietic differentiation from hPSCs. Schematic summary of reported strategies for hematopoietic differentiation from hPSCs. Human PSCs can be differentiated into hematopoietic cells (HSCs) by three strategies: OP9 coculture, direct differentiation, and transcription-mediated differentiation approach.

The advent of iPSCs has offered us remarkable access to investigate early human blood development and an infinite source of cells with clinical importance that can be used for immunotherapies. Furthermore, producing iPSC-derived HSCs and HE from patients with genetic disorders can allow for vital disease modeling and access to novel therapeutic methods via high throughput drug screening. Differentiation of hPSCs to hematopoietic cells has been accomplished using several strategies which include the monolayer culture of hPSCs, 3D cluster differentiation as embryoid bodies (EBs) or in a feeder-dependent coculture system (Figure 2). Numerous hematopoietic lineages which include erythrocytes, megakaryocytes and platelets, macrophages, dendritic cells, and lymphoid cells from hPSCs have been derived [83] and have been significant in contribution towards developing a model for human hematoendothelial development from hPSCs.

### 5.1. Coculture Differentiation System

The system involves coculturing undifferentiated hPSCs with murine bone marrow stromal cells in the presence of serum-containing media [84–86]. The hPSC/OP9 coculture system is a widely used hematopoietic differentiation approach which provides a major advantage because efficient hematopoietic differentiation from hESCs can be achieved within a short timespan (8-9 days) with the utilization of specific fetal bovine serum (FBS) and does not require additional cytokines [87]. Vodyanik et al. have shown that of the different murine bone marrow stromal cells tested, the OP9 cell line is the most efficient at inducing hematopoietic transition [88]. OP9 coculture can be used to obtain multipotent hematopoietic progenitors and mature cells including T [89, 90] and B lymphocytes [88, 91] and megakaryocytes [92]. However, there are several limitations to the stromal cell coculture system. Cell density of OP9 cells, size of the hPSCs colony, and FBS lots are the most important factors extremely essential for the efficiency of the hPSC differentiation in the OP9 coculture system. These limitations pose a challenge to understanding signaling pathways involved in the hematoendothelial transition during hPSC differentiation. Furthermore, the use of xenogeneic material in the system limits the therapeutic benefits of the system. Despite the establishment of xenogeny-free hPSC culture, hPSC-derived hematopoietic cells of clinical importance need to be generated using defined methods of differentiation. In addition, studies also showed that teratoma-derived hematopoiesis of iPSCs was significantly improved when coinjected with OP9 stromal cells compared to iPSCs alone. These studies revealed that isolating and reinjecting hematopoietic progenitors from hPSC-derived teratomas have shown multilineage engraftment potential [93, 94]. Despite the fact that HSCs were lower in numbers and myeloid lineage tendency was seen after secondary transplantation, these results demonstrated that hPSCs possessed potential towards differentiating into HSCs.

### 5.2. Direct Differentiation System

Another method of differentiation is the direct differentiation of hPSCs by culturing them in chemically defined medium with the sequential addition of specific morphogens, cytokines, and small molecules in order to promote hematoendothelial differentiation [95]. Directed differentiation has been carried out by using embryoid bodies (EBs), which are 3D aggregates, or by a monolayer, 2D-system culturing of hPSCs. Although these protocols rely on the use of serum in the media [11], serum-free media have been developed to be used in these protocols recently [96–98]. Exploiting different signaling pathways by using Wnt agonists and bone morphogenetic protein 4 (BMP4) in cultures to induce efficient mesoderm, VEGF to improve angiogenesis, and hematopoietic cytokine cocktails to increase hematopoiesis have been useful to improve the efficiency of hematopoietic differentiation of hPSC-derived EBs [25, 31, 97, 99]. The EB-based differentiation system also poses several limitations due to the complex nature of the EBs, variations between each EBs, and its relatively slow differentiation that restricts the use of this system [31, 100].

On the other hand, the two-dimensional method (monolayer culture) involves direct differentiation on ECM-coated plates. While some groups have used Matrigel ECM which is derived from mouse sarcoma cell line, to plate cells, others have discovered the use of human collagen IV, laminin, and fibronectin as efficient matrices to support induction of mesoderm and support hematoendothelial differentiation [101–104]. Additionally, Uenishi et al. developed a technique that can generate HSCs from a monolayer of hPSCs. Through molecular profiling studies, they found that tenascin C is expressed highly in over confluent OP9 stromal cells with higher hemato-inducing activity and demonstrated tenascin C's ability to promote the development of hematoendothelial progenitors [105]. This two-dimensional method which involves stage-specific addition of growth factors, small molecules and cytokines, decreases the differentiation time but increases the efficiency of hematoendothelial differentiation, making it a highly efficient method that is completely chemically defined [103–105].

### 5.3. Transcription Factor-Mediated Differentiation System

EHT and HSC emergence in AGM is controlled by combinatorial transcription factor interaction. In transcription factor-mediated conversion, a particular cell fate is activated by the exacted expression of key transcription factors. By using transcription factors that are vital in hematopoietic differentiation, multiple conversion approaches have been reported recently involving hPSCs [106–110]. During the transition period from mesodermal to hematopoietic lineages, the transcription factor Scl plays a significant role in early hematopoietic development [111]. Sandler *et al.* demonstrated that in the human system, overexpression of transcription factors like RUNX1, FOSB, SPI1, and GFI1 in HUVECs or adult dermal microvascular endothelial cells followed by coculture with AKT-activated endothelial cells induced definitive hematopoietic development or the HSC program [112]. Szabo *et al.* has reported that human fibroblasts overexpressing a single transcription factor OCT4 when transplanted into NSG recipients produced myeloid engraftment compatible with cord blood CD34^+^ cells and erythroid colonies expressing adult *β*-hemoglobin and lacking embryonic *ε*-hemoglobin [113].

In ES cell culture models, RUNX1 is recognized as the master regulator of EHT and its expression in the yolk sac progenitors has also shown to develop HE in the dorsal aorta and even a certain number of HSCs [114, 115]. The balance between RUNX1 and HOXA3 is important for the development of HE stage [116]. HoxA3 also upregulates the transcriptional factor Sox17 that plays an important role in specifying arterial and HSC emergence [116–118]. Earlier, a gain-of-function screening system was developed to determine the important transcriptional regulators of HE formation from human PSCs [107]. Based on this system, it was revealed that the enforcing expression of various combinations of transcription factors converted hPSCs into different hematopoietic progenitors; none of the factors could induce blood formation when used alone. The combination of transcription factor ETV2 and GATA2 led to the induction of CD43^+^ blood cells with panmyeloid potential, whereas the combination of TAL1 and GATA2 endowed cells with erythromegakaryocytic potential which involved a HE intermediate stage [107]. The hPSC-derived hematopoietic progenitors generated mature colonies in methylcellulose-based assays but were unable to engraft long-term in vivo [107]. Interestingly, Suknuntha et al. used modRNA expressing ETV2 or ETV2 and GATA2 to generate endothelial and CD34+CD43+ hematopoietic progenitor cells from HPSCs and nonhuman NHP, respectively [119].

Recently, it was shown that the multipotentiality of pluripotent stem cells and differentiation into various tissue types during embryogenesis can be controlled by sequential exposure to morphogens. Sugimura et al. performed modified morphogen-directed differentiation of pluripotent stem cells to generate hPSC-derived CD34^+^ cells, which were then subsequently enforced to express seven common transcription factors (ERG, HOXA5, HOXA9, HOXA10, LCOR, RUNX1, and SPI1) which are commonly detected in myeloid, B and T cell populations [110, 120, 121]. To determine their necessity in hematopoiesis, they transduced HE with these seven factors and engrafted them into irradiated mice. Efficient multilineage hematopoietic reconstitution in mice and the development of functional myeloid, B and T cell populations, was observed [110]. Although engraftment was possible with these genetically modified cells, they possessed contrasting functional and molecular traits compared to HSCs derived from cord blood. Concluding from these results, it is clear that the controlled expression of certain factors can generate HSC-like cells that are not fully functional shedding light on the importance of learning the mechanisms molecular regulators undergo in mediating definitive hematopoiesis. Nevertheless, there is promising evidence that the direct conversion of somatic cells into HSCs can be a feasible option for future clinical applications.

## 6. Various Stages of HSC Development during hPSC Differentiation

A thorough knowledge of the various stages of hematopoietic development and the mechanisms behind the regulation of induction and specification of hematovascular progenitors from hPSCs is important. At the moment, an extensive model of hematoendothelial development with hPSCs includes the use of OP9 stromal cell coculture and direct differentiation [31, 62, 105].

### 6.1. Mesoderm Stage

**Figure 3:**
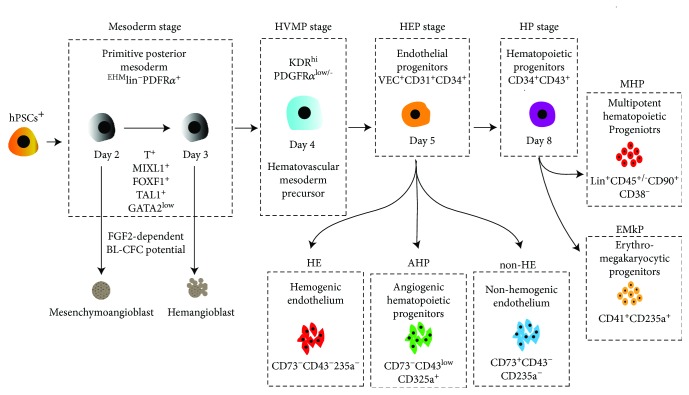
Established stages of hematopoietic development from hPSCs^+^. The primitive mesodermal precursors are capable of forming mesenchymoangioblast (MB) and hemangioblast (HB) in the presence of FGF2 [62, 122, 125]. Mesodermal commitment to angiohematopoietic development progressively leads to the formation of ^EMH^lin^−^KDR^bright^APLNR^+^PDGFRa^low/−^ hematovascular mesodermal precursors (HVMPs) [62, 89]. The HEP stage was identified based on the expression of the typical endothelial markers VE-cadherin, CD31, and CD34 and the absence of the panhematopoietic marker CD43 [62, 88]. HE cells were distinguished from non-HE cells based on the presence of CD73 expression [62, 132]. Initial hematopoietic progenitors arising from the VE-cadherin+ population show the presence of CD235a, low levels of CD43, and absence of CD41a expression. These cells can form hematopoietic colonies in the presence of FGF2 and retain their endothelial potential. These progenitors were labelled as angiogenic hematopoietic progenitors (AHPs) [62, 132]. Progressive hematopoietic development is identified by the appearance of CD43 expression, and all hematopoietic CFCs are accumulated in this fraction. Distinct subsets of CD43^+^ hematopoietic cells, including CD41a^+^CD235a^+^ erythromegakaryocytic progenitors and lin−CD34^+^CD43^+^CD45^+/−^ multipotent myelolymphoid progenitors, are also established [63, 88, 89, 105].

Induction of the primitive mesoderm is the first stage of hPSC differentiation which can be identified by the expression of mesodermal marker APLNR and KDR and a lack of expression of typical endothelial (CD31, VE-cadherin), endothelial/mesenchymal (CD73, CD105), and hematopoietic (CD43, CD45) markers [63, 122]. Hematoendothelial lineages that arise from the mesoderm have been specifically described as expressing KDR^+^ APLNR^+^ (Flk-1, VEGFR2, and CD309,) and PDGFRa^+^ (CD140a) [31, 62, 104] (Figure 3). Several studies utilizing the EB differentiation method have shown that the emergence of the primitive streak and appearance of the mesoderm populations are dependent on the bone morphogenetic protein 4 (BMP4), the fibroblast growth factor 2 (bFGF), as well as Nodal and WNT-*β* catenin signaling pathways [83, 97]. Several other studies have found that inhibition of GSK3*β* (a Wnt-signaling inhibitor) can induce mesoderm formation in PSCs from human and nonhuman primate [123, 124]. Sturgeon et al. demonstrated that early manipulation of WNT-*β*-catenin signaling can specify distinct primitive and definitive hematopoiesis waves that develop from separate mesoderm populations [31].

During the mesodermal stage of development, three different clonogenic progenitors with varying endothelial potential are formed. The first form is the mesenchymoangioblast (MB), which is defined as a precursor of endothelial and mesenchymal cells [122, 125, 126] (Figure 3). The second clonogenic progenitor is marked by the emergence of blast CFCs (BL-CFCs) [62, 63, 122] (Figure 3). BL-CFCs are commonly referred to as hemangioblasts (HB) because they consist of vascular and hematopoietic progenitors. Both MB and HB arise in coculture with OP9 or a direct differentiation system on days 2 and 3 of differentiation [62, 89, 105]. Both MB and HB potentials can be detected using colony-forming assay in serum-free clonogenic medium supplemented with FGF2 [122] (Figure 3). It was recently reported that overexpression of ETS1 during the mesodermal stage of development dramatically enhances the formation of arterial-type HE that express DLL4 and CXCR4 [127]. The last one is cardiovascular progenitors which have endothelial and cardiomyocyte potentials [128].

### 6.2. Hematovascular Mesoderm Precursor (HVMP) Stage

The next step of more advanced mesodermal commitment is associated with formation of lateral plate-like mesoderm cells, which are known as hematovascular mesoderm precursors (HVMPs) [62]. HVMPs arise in coculture with OP9 or in the direct differentiation system on day 4 of differentiation [62, 89, 105, 122]. The emergence of HVMPs can be detected based on high expression of KDR and low to no expression of PDGFR*α* in ^EMH^lin^−^APLNR^+^ cells, i.e., ^EMH^lin^−^KDR^bright^APLNR^+^PDGFRa^low/−^ phenotype. The development of the HVMP stage is mainly promoted by continued activation of the Wnt signaling pathway [101]. During the HVMP stage, expression of *TAL1*, *HHEX*, *LMO2*, *GATA2*, and *ETV2* genes associated with angiohematopoietic development are upregulated (Figure 3). HVMPs do not possess BL-CFC potential but are abundant in bipotential cells that can form hematoendothelial clusters when cocultured on OP9 and can therefore produce all myeloid progenitors [62]. Together, these results suggest that primitive hematopoietic potential can be detected within immature posterior mesoderm cells, whereas more mature and developing HVMPs generate blood cells with definitive characteristics. In the same line, recently, we demonstrated that HVMPs with definitive hematopoietic potential produce the highest numbers of T cells when cultured on OP9-DLL4 compared to other progenitors [89].

### 6.3. Hemogenic Endothelium Progenitor (HEP) Stage

Producing HEP populations from hPSCs is considered as a vital step progressing towards the genesis of blood progenitors, and this population can be identified by the expression of the typical endothelial marker VE-cadherin, CD31, and CD34 and the absence of the panhematopoietic marker CD43 [62, 99, 129]. VEC^+^ cells represent a heterogenous population which can be divided into 3 independent populations, HE, non-HE, and AHP (Figure 3). HE cells can be readily distinguished from non-HE cells based on the lack of CD73 expression in HE cells [62, 129]. These cells lack hematopoietic CFC potential but can form blood populations after culturing with stromal cells [62]. HEPs differentiated from hPSCs present the CD144^+^CD31^+^CD73^−^CD43^−^ phenotype. The non-HE cells are identified by the direct upregulation of CD73 [62] and under NOTCH signaling, HEP specify into DLL^+^ arterial HE and DLL4^−^ nonarterial HE [54]. Some studies have found that based on location, the population of non-HE cells can be further divided into CD73^med^CD184^+/-^DLL4^+^ arterial and CD73^hi^CD184^−^ venous endothelium populations. This separation between arterial and venous HE can be induced by altering various signaling pathways like mitogen-activated protein kinase (MAPK), NOTCH, and phosphoinositide 3-kinase (PI3K) pathways [130, 131]. In addition to these 2 populations, a third population named as angiogenic hematopoietic progenitor population (AHP) is identified by the CD144^+^CD31^+^CD73^−^CD43^+^ phenotype (Figure 3). These hematopoietic progenitors can develop into hematopoietic colonies in a FGF2-containing methylcellulose culture and also form endothelial sheets in endothelial-specific culture exhibiting their angiogenic potential [62, 132]. Culturing these endothelial subsets in arterial, venous, and lymphatic conditions revealed that AHPs are skewed towards lymphatic, HEPs towards arterial, and non-HEPs towards venous differentiation *in vitro*. These findings suggest that selection and enhancement of production of a particular EC subset may aid in generating desirable EC populations with arterial, venous, or lymphatic properties from hPSCs [132].

### 6.4. Multipotent Hematopoietic Progenitor (MHP) Stage

Significant progress has been made in the last two decades in understanding blood development from hPSCs. CD43 (leukosialin) has been reported to be the initial marker that specifies hematopoietic progenitors from endothelium in hPSC differentiation cultures [88], however, debated that hematopoietic progenitors expressing CD43 maybe of primitive lineage [31]. This issue paved way for the precise separation of CD43^+^ hematopoietic cells from preceding VE-cadherin (VEC)^+^CD43^−^ HEP progenitors. Currently at this stage, it is considered that advanced hematopoietic development occurs due to EHT, which is associated with the upregulation of CD43 expression and when all hematopoietic CFCs segregate into CD43^+^ fractions [88, 99]. The CD43^+^ subsets include lin-CD34^+^CD43^+^CD41^+^CD235a^+^ erythromegakaryocytic progenitor (E-MkP) and lin^−^CD34^+^CD43^+^CD45^+/-^ multipotent hematopoietic progenitors (MHPs) [88, 90, 99, 133] (Figure 3). The CD235a^+^CD41a^+^ cells are highly refined in erythromegakaryocytic progenitors that lack endothelial capacity. Shortly after the emergence of CD235a^+^CD41a^+^ cells, progenitors with broad lymphomyeloid capability and lin-CD34^+^CD43^+^CD45^−^ phenotype can be detected in hPSC cultures. The acquirement of CD45 phenotype by lin^−^ cells can be tracked to gradual myeloid engagement [88]. E-MKPs were essentially lacking T cell potential [89]. lin^−^CD34^+^CD43^+^CD45^+/-^ MHP cells can be characterized by myelolymphoid multilineage potential and the ability to be maintained and expanded in culture. MHPs have granulocyte-erythroid-macrophage-megakaryocyte colony-forming potential (CFC-GEMM) and T-lymphoid potential. MHPs can generate enucleated erythrocytes with *γ*- and limited *β*-globin expression as well. With specific treatment or addition of interleukin-3 (IL-3), interleukin-6 (IL-6), stem cell factor (SCF), and thrombopoietin (TPO) [62, 86–88], erythropoietin (EPO), Flt-3 ligand (FLT3L), interleukin-11 (IL-11), epithelial growth factor (EGF), insulin-like growth factor 1 (IGF-I), and insulin-like growth factor 2 (IGF-II) can promote HP maintenance and expansion in defined condition [31, 130].

## 7. NOTCH Signaling as the Master Regulator of Hematopoiesis

A clear understanding of the pathways involved during hematopoiesis is essential to clearly distinguish between primitive and definitive hematopoiesis. Signaling pathways play a vital role in cell development and specification that is also mainly defined by gene regulation [134]. While most of the cell signaling pathways have been demonstrated to be required for HSC formation, HSC specification requires signaling pathways that are nonessential for other hematopoietic waves. Based on studies, it was observed that the emergence of HSCs requires WNT [31, 135], BMP4 signaling [136, 137], NOTCH [15, 47, 54, 130, 138], VEGF [139], SCF [49, 140], and Hedgehog [141] signaling. Among all, the NOTCH signaling pathway has been extensively studied and has been shown to be critical during the onset of definitive hematopoiesis [54, 130, 138, 142]. Notch signaling is involved in lineage commitment, lateral inhibition between neighboring cells, and maintenance of homeostasis [143]. In mammals, key proteins involved in NOTCH signaling include four transmembrane NOTCH receptors (Notch 1-4) which are composed of an extracellular domain (NECD) and an intracellular domain (NICD), their associated Jagged1-2/Delta-like (DLL1, DLL3, and DLL4) ligands that vary in number across species [138]. It also includes enzymes that modify Notch ligands during activation (Mindbomb) and proteases that cleave activated receptors (gamma secretase/ADAM TACE) at the site 2 (S2) and site 3 (S3) to remove NECD from the rest of the receptor and to release NICD from the membrane, respectively. After translocation of NICD into nucleus, it interplays with the transcription factor complex, CSL (CBF-1/RBPjk, SuH, and LAG-1), to expulse corepressors and help the coactivator mastermind to trigger transcription of NOTCH target genes [143, 144]. *In vitro*, Notch signaling can be activated by coculturing cells with OP9 stromal cells that express the Notch ligands or by coating immobilized Notch ligands to the cell culture plates [54, 83, 145].

Recent evidence suggests that NOTCH signaling is explicitly required at the EHT stage of development and NOTCH dependency is a hallmark characteristic of definitive hematopoiesis [54, 130]. Notch signaling plays an important role in different stages of HE development, from arterial specification [131] to T-lymphocyte development [146]. Through several transgenic mouse studies, it has been proven that the primitive wave of hematopoiesis is NOTCH-independent [31, 99, 147] while the definitive wave of hematopoiesis is specifically NOTCH-dependent [147–149]. It has also been shown through mouse knockout studies that Notch activation is essential for the arterial specification of endothelial cells during vasculogenesis [150, 151]. Recent studies have shown that the activation of Notch signaling in the early HE specify them to VEC^+^CD43^−^CD73^−^DLL4^+^ arterial-type HE which is dependent on NOTCH for EHT and produce definitive lymphomyeloid and erythroid cells [15, 54]. The idea that Notch mediated arterialization of HE is an important stage for establishing the definitive hematopoietic program that sheds light on an arterial specification-dependent model of definitive hematopoietic development [15].

## 8. Outlook and Concluding Remarks

With significant developments in understanding embryonic hematopoietic development, there have been many approaches towards simulating developing systems to hematopoietic differentiation. Considering that the first HSCs, hESCs, and iPSCs were derived or discovered in 1961, 1998, and 2007, respectively, there have been substantial advancements in the involvement of stem cells in hematopoietic research. With understanding of the hematopoietic transition and various lineages, hESCs and iPSCs have been successfully used to produce almost all types of mature blood cells, although more consistent and efficient models are desired for this achievement. Additionally, the important understanding that HE and the EHT are vital for hematopoietic development and the observation of HE in developing hPSC cultures is important to further improve our models and protocols for definitive hematopoiesis.

With most of the stepwise process of hematopoietic differentiation generally understood, there are still shortcomings in the knowledge of certain signaling pathways and conditions of certain steps leading to various blood cell types. Though, with the clear evidence of hematopoietic specification resulting from the EHT or HE lineage, defining and establishing these conditions in hPSC models provide opportunity for differentiating hPSCs into HSCs and mature blood cells with long-term engraftment and self-renewing potential. These advances can really bring us closer towards dealing with clinical applications and applying such development techniques and engraftment of HSCs or various hPSC-derived blood populations towards therapies for blood-related disorders.

Continuing further, more *in vivo* studies with various model organisms on hematoendothelial development in sites of interest that include the AGM, yolk sac, or arterial and nonarterial sites will pave the way to clarifying our current knowledge of the hematopoietic transition and develop ideal environmental conditions to produce efficient *in vitro* hPSC models of hematopoietic differentiation. Commendable research has been accomplished since the finding of both primitive and definitive waves of hematopoiesis.

Disease treatment has been revolutionized by the clinical benefits of stem cell transplant. Further understanding of hematopoiesis and replicating the developmental process *in vivo* can revolutionize the future of regenerative medicine.
